# Lung Cancer Screening in Family Members and Peers of Patients With Lung Cancer: Protocol for a Prospective Cohort Study

**DOI:** 10.2196/58529

**Published:** 2025-03-28

**Authors:** Isabelle Pitrou, Adriano Petrangelo, Charlotte Besson, Carmela Pepe, Annika Helen Waschke, Jason Agulnik, Anne V Gonzalez, Nicole Ezer

**Affiliations:** 1 Centre for Outcomes Research and Evaluation (CORE) Research Institute McGill University Health Centre Montréal, QC Canada; 2 Division of Respiratory Medicine McGill University Health Centre Montréal, QC Canada; 3 Division of Respiratory Medicine Jewish General Hospital Montréal, QC Canada

**Keywords:** lung cancer, low-dose CT, chest tomography, lung cancer screening, patient advocacy, early detection of cancer, referral and consultation, cohort study, patient empowerment, patient experience

## Abstract

**Background:**

Low-dose computed tomography (LDCT) screening is promising for the early detection of lung cancer (LC) and the reduction of LC-related mortality. Despite the implementation of LC screening programs worldwide, recruitment is challenging. While recruitment for LC screening is based on physician referrals and mass advertising, novel recruitment strategies are needed to improve the enrollment of high-risk individuals into LC screening.

**Objective:**

We aim to identify whether patients with LC can act as advocates to enroll their family members and close contacts into LC screening and whether this strategy increases screening uptake at the population level.

**Methods:**

We designed a prospective cohort study comprising 2 cohorts constituted between June 2023 and January 2024 with a prospective follow-up of 18 months. Patients with LC (cohort 1) are approached at clinics of the McGill University Health Centre, educated on tools for communicating with family members and close contacts about the benefits of LC screening, and invited to refer their close ones. Referred individuals (cohort 2) are directed to this study’s web-based questionnaire to assess their LC risk score with the PLCOm2012 (Prostate, Lung, Colorectal and Ovarian Cancer Screening Trial) prediction model. Individuals meeting the eligibility criteria for LC screening (PLCOm2012 score ≥2% and aged 55-74 years) are directed toward the Quebec LC screening program. Data collected include sociodemographic characteristics, health literacy and smoking status (all participants), patient activation (cohort 1), perceived risk of LC, and generalized anxiety at baseline and at 28 days (cohort 2). LDCT completion within 18 months from referral is assessed from health records. Focus groups will identify the barriers and facilitators in the uptake of LC screening and preventative behaviors based on perceived genetic and clinical LC risks. The primary outcomes are the number of referred participants per survivor of LC and the mean risk of LC of the referred population based on PLCOm2012 scores. The secondary outcomes are the proportion of (1) participants eligible for LC screening; (2) participants eligible for screening who complete LDCT screening within 18 months of referral from a survivor of LC; (3) participants showing interest in genetic testing to inform LC risk; and (4) participants showing interest in a smoking cessation program. Multivariable logistic regression will identify the predictive factors of being referred for LC screening. PLCOm2012 scores will be compared for referred participants and controls from the provincial LC screening program.

**Results:**

Overall, 25 survivors of LC and 84 close contacts were enrolled from June 2023 to January 2024, with followed up through July 2025. The results are expected by the end of 2025.

**Conclusions:**

We describe an approach to LC screening referral, leveraging patients with LC as advocates to increase screening awareness and uptake among their family and peers.

**Trial Registration:**

ClinicalTrials.gov NCT05645731; https://clinicaltrials.gov/ct2/show/NCT05645731

**International Registered Report Identifier (IRRID):**

DERR1-10.2196/58529

## Introduction

### Background

Almost three-quarters of lung cancers (LCs) are diagnosed at stages III and IV with poor overall survival [1,2]. LC remains the cancer with the highest mortality rate, with high rates of comorbidities also [3,4]. The projected increase of LC burden in Canada calls for improving its early detection and preventative strategies [5]. Low-dose computed tomography (LDCT) screening of high-risk individuals is promising for early detection and reduction of LC-related mortality [6]. With more than 50,000 high-risk individuals enrolled, the pioneering National Lung Screening Trial has shown a 20% reduction in LC-related mortality with LDCT screening compared to standard chest radiography [7]. Evidence supporting this screening strategy was further supported by the NELSON (Nederlands-Leuvens Longkanker Screenings Onderzoek Trial), Pan-Canadian Early Detection of Lung Cancer Study, and UK Lung Cancer Screening Trial [8,9], leading to recommendations for screening high-risk individuals and the implementation of LC screening programs in North America and Europe [10].

The efficiency of LC screening programs relies on the optimization of the risk-benefit ratio and ensuring high participation rates within individuals with a higher risk of LC. Efforts to improve screening uptake within these groups are necessary as the preliminary uptake rates from pilot programs in the United States, Canada, and Europe were suboptimal, with only 4% to 13% of eligible individuals enrolling in LC screening [11,12]. Recent scoping reviews identified the barriers to LC screening uptake, with the most important barriers being the lack of awareness of screening programs, perceived smoking-related stigma, socioeconomic difficulties, and fear of receiving a cancer diagnosis. Furthermore, individuals facing these barriers have concomitantly higher risk of LC [13,14]. For example, in Canada, the highest rates of LC are clustered among individuals living in rural areas with lower socioeconomic status, who also have lower access to screening programs for those geographical and socioeconomic reasons [15,16]. Strategies to reach those most socially disadvantaged groups are thus imperative to guarantee the efficiency of LC screening.

Survivors of LC are empowered with valuable knowledge on their disease’s trajectory, from diagnosis to treatment. By sharing their stories within their communities, they can empower other individuals in similar health states. Due to a combination of both genetic and shared risk factors such as smoking or exposure to radon, individuals with a first-degree relative with a history of LC have a 2- to 3-fold higher risk of LC compared to the general population [17]. In a qualitative study, among individuals eligible for LDCT screening per the US Preventative Services Task Force, Roth et al [18] demonstrated that having friends or family members being treated for LC was a major motivator for being screened themselves.

So far, no previous studies have examined the impact of using survivors of LC as sources of education and referral for peers and family members who may be eligible for screening. We hypothesize that survivors of LC would be willing to refer family members or peers to LC screening, leading to a positive impact on the uptake of both the preventative and screening behaviors of the referred population. This study thus aims to determine the feasibility and acceptability of referral to LC screening through patients with LC referring their close ones to LC screening and to assess the early impact of this novel recruitment strategy on the uptake of LC screening and the patients’ outcomes.

### Objectives

We aim (1) to examine if patients with LC are willing to refer family members or close contacts to LC screening and if referral is associated with increased patient activation; (2) to examine if targeted enrollment of family members or close contacts of patients with LC for LC screening leads to increased engagement of individuals at higher risk of LC compared to referral through usual care; and (3) to examine the barriers and facilitators on the uptake of preventative strategies and LC screening, based on perceived genetic and clinical LC risks.

## Methods

### Study Design

This is a prospective cohort study of 2 separate cohorts.

### Participants Eligibility and Recruitment

#### Cohort 1

**Figure 1:**
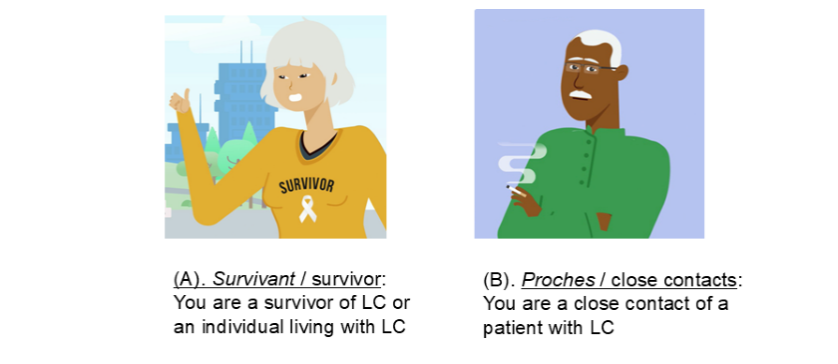
Screenshot of the web page for survivors of lung cancer and their close contacts. LC: lung cancer.

The first cohort includes biopsy-proven patients with LC aged older than 18 years at various stages in their disease trajectory, including those undergoing clinical surveillance. Participants in cohort 1 are recruited into this study either through discussion with trained research staff approaching them in the clinical settings or through posters or pamphlets available for viewing in the waiting areas of the clinics. The clinical settings where participants are recruited include the thoracic surgery clinic, pulmonary oncology clinic, pulmonary procedural suites, and chemotherapy infusion centers located at the McGill University Health Centre, one of the largest university hospitals in Montreal, Canada. Recruitment discussions and materials invite these patients to access this study’s website [19]. The web page section directed at participants in cohort 1 (Figure 1A) includes educational material on the rationale for LC screening and the methods on how to approach and discuss LC screening with peers and family members.

Patients interested can consent and enroll through this study’s website using digital or paper forms. Participants are invited to provide contact information (email or phone number) for the close ones they wish to refer for inclusion in the second cohort of our study. They are also advised to inform their close contacts that they shared their details with the research team and that their informed consent will be mandatory before participation.

#### Cohort 2

The second cohort includes participants being referred for inclusion into our study by the participants of cohort 1. Referred participants should be aged older than 18 years. Individuals with a personal history of LC are excluded. Referred participants are contacted by our research team through emails, phone calls, or through a recruitment pamphlet mailed for those without internet access. Participants are encouraged to access our study’s website [19]. The web page section directed at cohort 2 (Figure 1B) provides general information on LC, its epidemiology, information on LC screening programs and the risk-based eligibility, and information on risk mitigation strategies such as smoking cessation and home radon testing. The enrollment of referred participants is also carried out with a submittable digital form available on this study’s website. Participants lacking the digital literacy to enroll by themselves are contacted by the research team to enroll over the phone or through mailed material.

Once enrolled, participants have their 6-year risk of LC estimated with the PLCOm2012 (Prostate, Lung, Colorectal and Ovarian Cancer Screening Trial) risk model [20]. The risk prediction PLCOm2012 model has been extensively validated in previous studies [21,22]. The PLCOm2012 model has also shown adequate properties to discriminate patients with LC in a large cohort of Quebec smokers [23]. Moreover, data from the International Lung Screening Trial have shown that the PLCOm2012 model was more efficient compared to the US Preventative Services Task Force 2013 criteria to identify high-risk individuals to enroll into LC screening programs [24]. Participants who meet the eligibility criteria for the provincial LC screening program (ie, aged between 55 and 74 years and PLCOm2012 score ≥2%) are directed toward the provincial LC screening program to complete LDCT.

### Website and Educational Material

This study’s website [19] and educational material were developed based on 3 main pillars: accessibility, comprehensibility, and inclusivity. First, we succinctly presented information to account for the health literacy level of the targeted population. Individuals at high risk of LC and with lower education levels are significantly less likely to enroll for LC screening and engage in harm-reduction programs [25], underlining the importance of presenting information comprehensible for all. This study’s website content was developed in collaboration with current patients with LC with written material created to be comprehensible at a 5th-grade reading level and above. Second, audiovisual contents are more easily comprehensible and with lower decisional conflict when used as a patient decision aid compared to information presented in textual format [26]. The audiovisual format is also more inclusive of participants with very low literacy. In collaboration with the digital media company Tactica Interactive [27], videos were produced to include the textual information of the website in audiovisual format. Lastly, for participants interested in enrolling in this study but lacking the digital literacy to enroll by themselves, information is provided on this study’s website to contact this study’s research staff (email address and telephone number).

### Outcomes

The primary and secondary outcomes are summarized in Table 1.

**Table 1:** Study outcomes.

Outcomes			Methods
**Primary outcomes**			
	Number of referred participants per individuals with LC^a^ (survivors of LC)	Questionnaire: cohort 1	
	Mean risk of LC in the referred population	Questionnaire: cohort 2 PLCOm2012 model	
**Secondary outcomes**			
	Proportion of participants eligible for the Quebec provincial LC screening program	Questionnaire: cohort 2 PLCOm2012 model	
	Proportion of participants eligible for screening who complete LDCT^b^ screening within 18 months from referral	Provincial health data	
	Proportion of participants who demonstrate interest in undergoing genetic testing to inform their LC risk	Questionnaire: cohort 2	
	Proportion of individuals who demonstrate interest in a smoking cessation program among referred participants who are current smokers	Questionnaire: cohort 2	
	Barriers and facilitators in the uptake of LC screening and preventative strategies, based on perceived LC risk	Focus groups: cohort 1 and cohort 2	
	Number of visitors on the study’s website for referred participants	Google analytics data	

^a^LC: lung cancer.

^b^LDCT: low-dose chest tomography.

### Data Collection

#### Overview

Data are collected using the REDCap (Research Electronic Data Capture; Vanderbilt University) platform [28]. Participants are sent links to complete the web-based questionnaires.

Baseline data collected for all participants include sociodemographic characteristics (age, sex, education level, and ethnicity), health literacy, and smoking status. Health literacy is assessed with the 3-item Brief Health Literacy Screen [29] questions: “How often do you have someone help you read hospital materials?” “How confident are you filling out medical forms by yourself?” and “How often do you have problems learning about your condition because of difficulty understanding written information?” rated on 5-point Likert scales.

#### Cohort 1

For participants with LC (cohort 1), the phase of LC trajectory (investigative, treatment, or surveillance), tumor stage, performance status (as defined per Eastern Cooperative Oncology Group performance status grading), and their relationship to the referred participants are collected. The degree of self-empowerment they experienced by referring peers and family members for LC screening is assessed using the 13-item Patient Activation Measure (PAM) questionnaire [30]. The 13-item PAM questionnaire is a validated instrument to measure patient knowledge, skills, beliefs, and confidence for self-managing health. Patient activation stands as an important pillar of patient-centered care [31], and evidence has shown that being engaged and active in one’s own care is linked to better outcomes [32]. For each participant with LC, the number of unique web page visits is quantified using Google Analytics, and the number of referrals per participant with LC is collected. For participants who refer family members only, supplementary questions are asked to explore the potential barriers: “I don’t feel comfortable speaking with friends or close contacts about their health”; “I did not want my friends or close contact who smoke or used to smoke feel judged about their smoking”; “I did not want my friends to worry about their risk of LC”; “Speaking about LC screening and/or LC in general brings up negative emotions for me”; “I don’t see or speak with my friends or close contacts very often”; and “This study did not help me feel more comfortable discussing the potential benefits of LC screening.”

#### Cohort 2

For referred participants (cohort 2), the web-based questionnaire collects their known personal history of chronic obstructive pulmonary disease, prior exposure to radon or asbestos, smoking habits, and nicotine dependence using the Fagerström Tolerance questionnaire [33]. Data are used to calculate their PLCOm2012 six-year LC risk. The perceived risk of developing LC is assessed by asking “Compared with others your age, what do you think your chances are of being diagnosed with LC during your lifetime?” rated on a 5-point Likert scale. We also assess whether referred participants believe their risk is high or low due to genetics or smoking exposure and which they think is more important in increasing their lifetime LC risk. Generalized anxiety is assessed at baseline using the General Anxiety Disorder 7-item questionnaire (GAD-7) [34], and a question asks if they believe their anxiety is associated with their perceived risk of LC. We repeat the GAD-7 at 28 days after enrollment to assess the impact of the LC screening risk assessment on anxiety levels. Patients will be asked to give their consent to access their provincial health records within the 18 months following their enrollment in this study. Extracted data will consist of whether these participants underwent LC screening via LDCT within 18 months after enrollment. No data about the results of the LDCT imaging will be extracted.

#### Both Cohorts

Focus groups are conducted to explore the perceptions of LC risk (perceptions of genetic risk based on polygenic scores vs perceptions of clinical risk based on PLCOm2012 scores) and their associations with engagement in LC screening and preventative behaviors (such as smoking cessation, radon measurement, and remediation). Qualitative questionnaires and clinical scenarios are presented to this study’s participants of both cohorts to explore these themes and elucidate the most prevalent barriers and facilitators toward engagement in preventative behaviors and screening programs. Focus groups will be audio visually recorded and transcribed in verbatims.

### Statistical Analysis

#### Quantitative Analysis

Participants with LC who refer at least one peer for participation will be compared to those who did not refer a peer for participation. To identify the predictive factors of referral, multivariable logistic regression will be used, adjusting for age, sex, educational attainment, health literacy, PAM score, and phase of LC trajectory. The dependent variable in the logistic regression will be referral by patients with LC. Using logistic regression, we will also examine if increased patient with LC activation is associated with increased odds of referral. Chi-square tests and 2-tailed *t* tests will be used for comparisons of categorical and continuous variables.

Referred participants who are eligible for LC screening will be considered as cases. A control group will be selected from patients who were either self-referred or referred by their primary care physician to the Quebec LC screening program and matched by age (1:1). To determine if participants in cohort 2 have a higher risk of LC than the controls, the mean PLCOm2012 scores will be compared using 2-tailed *t* tests in our cohort and in the Quebec LC screening program. The predictive factors of being eligible for LC screening and whether being referred by survivors of LC is associated with increased odds of being eligible will be examined using multivariable logistic regression. We will account for clustering among individuals referred by the same family member using a random effect model.

Spearman coefficients will be calculated to assess the correlations between the actual risk and the perceived risk of LC. Kruskal-Wallis tests will be used to compare actual and perceived risks of LC with different levels of anxiety. Odds ratios and their 95% CIs will be reported. All statistical analyses will be conducted using R (version 4.2.0; R Foundation for Statistical Computing).

#### Qualitative Analysis

Focus groups consisting of both cohorts will be conducted. For referred participants, the association between their perceived risk of LC based on both their genetic perceptions of risk and their actual clinical risk of LC and undertaking preventative behaviors and screening will be explored.

First, focus groups consisting of both cohorts will be audiovisually recorded and transcribed in verbatims. Responses will be labeled with descriptive codes by 2 independent analysts using NVivo (Lumivero). Interrater agreement and κ coefficients will be calculated to assess intercoder reliability and consensus reached through comparison and discussion within the panel group. The second stage will involve constant comparison, where codes and their content are compared across interviews to discern common and divergent themes. In the final stage, the data will be organized by searching for patterns, variations, and relationships between themes to characterize the entire dataset.

### Sample Size

Preliminary data from the provincial LC screening program indicate that 40% of patients either referred to the program by their primary care physician or via self-referral meet the eligibility criteria for LC screening. The McGill University Health Centre receives approximately 800 new patients with LC annually, and the provincial LC screening program receives around 100 referrals each month. With a sample size of 194 referred participants, we will be able to detect a 20% difference in the rates of referral, with a power of 80% and an α value of .05.

### Ethical Considerations

This study has been approved by the McGill University Health Centre’s Research Ethics Board (MP-37-2023-9041). All participants will provide their informed consent before participation and will be informed that they can withdraw from this study at any time. Informed consent is obtained in either the English or French language through (1) internet-based web forms, (2) phone calls with trained research staff, or (3) completing consent forms in person when approached in one of this study’s clinics. To ensure participants fully understand this study’s goals when they enroll, three simple comprehension questions are asked being: (1) if this study involves medications (false), (2) if participants will be asked to complete questionnaires (true), and (3) whether they will be asked to refer family and friends (true). If participants have any questions or concerns regarding this study, they can contact our research team via telephone or email at any time (contact information is available on this study’s website, pamphlets, and posters).

The REDCap platform used to collect data conforms to the General Data Protection Regulation and Canadian privacy legal and security standards. Access to the REDCap platform is secured with private log-ins and 2-factor authentication. The REDCap database is securely hosted on the Research Institute of the McGill University Health Centre server. No information will be released to unauthorized third parties without prior written approval of the participant except as necessary for monitoring by public health authorities or our institutional research board. Participants will not receive compensation for their enrollment in this study. Those participating in focus groups will be reimbursed on an hourly basis by the Research Institute of the McGill University Health Centre.

## Results

Enrollment in the cohorts was conducted from June 2023 to January 2024, with participants being followed up through July 2025. Overall, we have enrolled 25 survivors of LC who have referred 84 of their close contacts to this study. The results of this study are expected to be reported at the end of 2025 through publications in peer-reviewed journals and presentations at relevant national and international conferences.

## Discussion

### Principal Findings

This paper describes the protocol of a pilot study examining the acceptability, feasibility, and impact of an innovative strategy of referral using patients with LC as advocates to increase the uptake of LC screening among individuals with high risks of LC. Increasing the uptake of LC screening for high-risk individuals is crucial as early detection of LC with LDCT has proved effective in reducing LC-related mortality by 20% and all-cause mortality by 7% [9]. LC screening with LDCT presents unique barriers that hamper the implementation and efficiency of these programs at the population level. In our study, survivors of LC receive education aimed at increasing awareness and enrollment in LC screening programs. We postulate that encouraging survivors of LC to empower and refer their close ones can not only have a positive effect on uptake rates for LC screening but can also improve patient activation and the psychosocial and clinical outcomes of patients with LC themselves.

LC is clustered among individuals with lower socioeconomic status who often also reside in rural areas [15,16,35,36]. The low screening uptake within these subpopulations has been well-established through prior LC screening programs [37]. Determining eligibility for screening is unique to LC screening and requires more extensive shared decision-making discussions between provider and patient compared to other cancer screening programs. As a result, primary care physicians need to dedicate a significant portion of a clinical visit to an LC screening discussion, which can be time-consuming. These subpopulations often face limited access to health care services while concomitantly having a high burden of clinical comorbidities. Thus, health care visits are both limited in frequency and in time as the other comorbidities may require further investigation. A recent study has shown that 67% of primary care physicians would not engage in LC screening discussions if they expected that the discussion would exceed 8 minutes [38]. Placing the referral burden entirely on primary care physicians appears to be unrealistic. These subpopulations with a higher risk of LC may benefit from this approach of encouraging survivors of LC to raise awareness within their social networks and reduce the burden placed on the providers servicing these groups.

As opposed to other cancer screening programs, such as those for breast, colon, and cervical cancers, there is a robust causal association between LC and “self-inflicted” behaviors in individuals at high risk [39]. This perceived smoking-related stigma has a negative psychosocial impact on high-risk patients, leading to self-blame, guilt, and hesitance in discussing their risk of LC with health care professionals and peers [40]. Smoking-related stigma impacts the patient-provider relationships as patients who smoke tobacco are often hesitant to provide an accurate disclosure of their smoking status to their provider and often demonstrate avoidance to discuss topics such as smoking cessation and investigations for ongoing respiratory symptoms. Prior evidence has also consistently shown that this smoking-related stigma leads to delays in seeking medical evaluation when experiencing the presenting symptoms of LC and limits patient involvement in treatment and survivorship care [41]. In turn, this smoking-related perceived stigma leads to hesitancy in seeking enrollment into LC screening programs. Ali et al [42] explored the barriers among high-risk individuals from enrolling in the UK Lung Cancer Screening Trial and determined that current smokers were significantly less likely to enroll in LC screening than exsmokers or never smokers. Compared to nonsmokers, current smokers were more fatalistic and less likely to consider LDCT for LC screening and also less likely to believe early detection would improve their chances of survival [43]. When exploring methods to mitigate the burden of smoking-related stigma, it emerges that current smokers experience the most empathy and can express their emotions more comfortably within support groups made up of other current smokers with similar “lived experiences” [44]. Using preexisting relationships as a forum for open communication and advocacy can therefore be fruitful. Perceived risk of LC can be an important motivator to help balance the negative effects of both practical and emotional barriers and engage in LC screening programs. Interestingly, the perceived risk of LC was positively correlated to the estimated risk of LC in participants from the Pan-Canadian Early Detection of Lung Cancer Study [45]. Furthermore, the perceived risk assessment of LC was associated with higher self-referrals, implying that encouraging individuals to both acknowledge and assess their own perceived risk of LC can be a significant motivator to enroll in screening programs. Using preexisting interpersonal relationships between survivors of LC and close contacts can be fruitful in establishing a forum of open communication to share lived experiences.

This study also aims to show that empowering survivors of LC to engage and motivate close contacts in LC screening and harm-reduction initiatives may have a positive impact on their own clinical outcomes and survivorship. In a recent qualitative study among survivors of LC, the concept of “passing it on”—referring to survivors’ experience with becoming and acting as educators for others—was identified as an important way to address their personal perception of the stigma of LC [46]. Survivors of LC placed significant value on the need to share their stories with their communities, to help family members learn about LC and navigate the health care system. In engaging in a conversation with close contacts regarding LC, they can express how the disease impacted their own well-being from the psychosocial stressors it placed on their mental health, the consequences on their interpersonal relationships, or the physical problems they face daily. Expressing these emotions to their social network may help them feel heard and understood, and in turn, their social network may be more aware and caring of the survivor’s well-being and needs.

### Limitations

This study should be interpreted considering the following limitations. First, the recruitment is conducted in clinical settings based on patients receiving in-person care, either through approaching patients with LC directly or via self-referral to our study’s website after viewing study-related posters or pamphlets in the waiting areas of clinics. Patients who receive telemedicine follow-up care (such as teleconsultations with health providers and real-time counseling) are then less likely to enroll in this study. This recruitment strategy may introduce a selection bias with a selection of participants in better health states who can participate in this study, living closer to this study’s urban health care center, or with a larger social network that supports them in their travel to this study’s site. However, this bias appears limited as telehealth for LC care remains marginal at our institution, as also stated in a review of telehealth in LC during the COVID-19 pandemic [47].

Second, when potential participants are approached in person or view our study’s recruitment material in the clinics, those accompanying them are exposed to the same discussion and material. Therefore, the referred cohort may be biased to preferentially include the first-degree relatives of survivors of LC as these are the close contacts most likely to accompany patients to their clinical appointments. Friends or more distant relatives of survivors of LC may ultimately be underrepresented in the referred cohort.

To finish, this study is conducted among a cohort of residents in Quebec, where public health care expenses are covered for citizens. The generalizability of the results is limited to similar populations and health care systems, and caution is needed when generalizing results to other Canadian provinces or countries.

### Future Directions

Primary care health providers and mass advertising are the current methods of recruitment to LC screening programs. In Canada and the United States, most participants who are eligible for LC screening are being referred to screening programs by a health provider. Barriers related to health care services access then limit the uptake of LC screening, notably in Canada, where approximately 15% of patients are unattached to a regular primary care provider [48]. This lack of providers per population is even worse in Quebec and is expected to worsen with the current population ageing. Developing new strategies to identify and enroll high-risk individuals into LC screening programs is crucial to ensure the success of implementing those programs and, in the end, to improve the patients’ outcomes.

### Conclusion

Early diagnosis of LC can improve survival rates, and strategies are needed to engage high-risk individuals in LC screening. We believe that patient advocacy has an important value and could be harnessed to identify high-risk individuals to participate in LC screening. Patients with LC have a unique role to play as advocates, and by sharing their lived experience, they could improve the motivation and engagement in LC screening and preventative strategies for their close ones. This study will provide evidence on the feasibility, acceptability, and early impact of this novel referral strategy. The results will be of interest for public health programs and policies, as well as for clinicians, patients facing LC, and their close ones.

## Abbreviations

GAD-7: General Anxiety Disorder 7-item questionnaire
LC: lung cancer
LDCT: low-dose computed tomography
NELSON: Nederlands-Leuvens Longkanker Screenings Onderzoek Trial
PAM: Patient Activation Measure
PLCOm2012: Prostate, Lung, Colorectal and Ovarian Cancer Screening Trial
REDCap: Research Electronic Data Capture
